# Analysis of factors influencing the intravertebral shell phenomenon after posterior reduction internal fixation of thoracolumbar fracture: a retrospective study

**DOI:** 10.1186/s12891-024-07168-9

**Published:** 2024-01-10

**Authors:** Yao Fang, Sining Zhang, Yuchao Ye, Kongning Chen, Guangfeng Ling, Qing Wang, Wugui Chen, Chengzhao Liu

**Affiliations:** 1 Department of Clinical Medicine, Faculty of Clinical Medicine, Fujian Medical University, Fuzhou, PR China; 2 Mindong Hospital Affiliated to Fujian Medical University, Ningde, PR China

**Keywords:** Thoracolumbar fracture, Osteoporosis, Pedicle screw-rod system, Intravertebral shell phenomenon, Risk factors

## Abstract

**Study design:**

A retrospective study.

**Purpose:**

The study objectives were as follows: 1) to analyze the factors influencing the occurrence of the intravertebral shell phenomenon (ISP) after thoracolumbar spinal fracture surgery and the evolutionary outcome of this phenomenon; and 2) to make recommendations for the clinical prevention and treatment of ISP.

**Methods:**

We retrospectively analyzed 331 patients with single-segment fractures of the thoracolumbar spine treated with internal fixation via a pedicle screw-rod system. Univariate and multivariate logistic regression were used to analyze factors influencing ISP.

**Results:**

A total of 260 patients (78.5%) developed ISP after surgery. Reduced bone mineral density, screw insertion depth, degree of vertebral body injury, and excessive vertebral body spreading were significantly associated with the occurrence of ISP (*P* < 0.05). A total of 166 of the 260 patients were reviewed via CT at 1 year postoperatively. Among them, 104 patients (62.6%) showed shrinkage or healed vertebral cavities, and 62 patients (37.4%) showed enlarged vertebral cavities or collapsed endplates.

**Conclusion:**

In clinical management, surgeons need to focus on risk factors for ISP, which include decreased bone density, preoperative vertebral overcompression, intraoperative vertebral overextension, screw insertion depth, and the degree of vertebral repositioning. At the 1-year postoperative follow-up, some of the vertebrae with ISP failed to heal or even showed vertebral cleft enlargement, which would affect the stability of the internal fracture fixation device and the quality of the patient's daily life.

**Supplementary Information:**

The online version contains supplementary material available at 10.1186/s12891-024-07168-9.

## Background

Thoracolumbar segment fractures are among the most common types of spinal fractures in clinical practice [1, 2] and account for approximately 60–70% of all spinal fractures. This is mainly attributed to their special biomechanical and anatomical characteristics [3]. The choice of treatment option for neurologically asymptomatic thoracolumbar fractures has not been fully clarified. A systematic review showed little difference in efficacy between surgical and nonsurgical treatment at ≥ 6 months for neurologically asymptomatic thoracolumbar burst fractures [4]. The current general principle of surgical treatment is that if there is spinal cord compression or neurological symptoms, open reduction and internal fixation with spinal canal decompression is generally chosen. If there is no neurological injury and the injury is mild, percutaneous pedicle screw fixation is usually chosen [5–7]. Many years of clinical experience have confirmed good clinical results using these methods, but there are still some problems to be discussed and studied.

Combining previous literature and our clinical observations, we have found that injured vertebrae treated with internal fixation devices frequently exhibit imaging features of intravertebral clefts [8–10]. Such a cleft usually manifests as a vertebral bone defect or vertebral body fracture. On CT, the main manifestation is an irregular low-density shaded area in the injured vertebral body, which forms a bone defect area resembling an eggshell-like parcel. We refer to this postoperative vertebral body defect phenomenon as the intravertebral shell phenomenon (ISP). Additionally, we have found that at 1 year after surgery, the area of bone defects in some of the vertebrae with ISP have further expanded and could even led to the collapse of the adjacent endplates, resulting in delayed or nonhealing of the fracture, internal fixation instability, and chronic back pain. Although previous studies have reported the development of such phenomena during the conservative treatment of fractures, little attention has been given to vertebral body changes after the surgical treatment of thoracolumbar fractures [8, 9, 11]. Most currently available studies on ISP are limited in scope to imaging characteristics and lack data on the early prevention, treatment, and evolution of this phenomenon. In this study, we present the occurrence of ISP in patients with thoracolumbar fractures treated with internal fixation via pedicle screws, as well as the factors that influence the outcome of the evolution of ISP at 1 year postoperatively, to provide a reference for the prevention and treatment of this phenomenon.

## Materials and methods

**Fig. 1:**
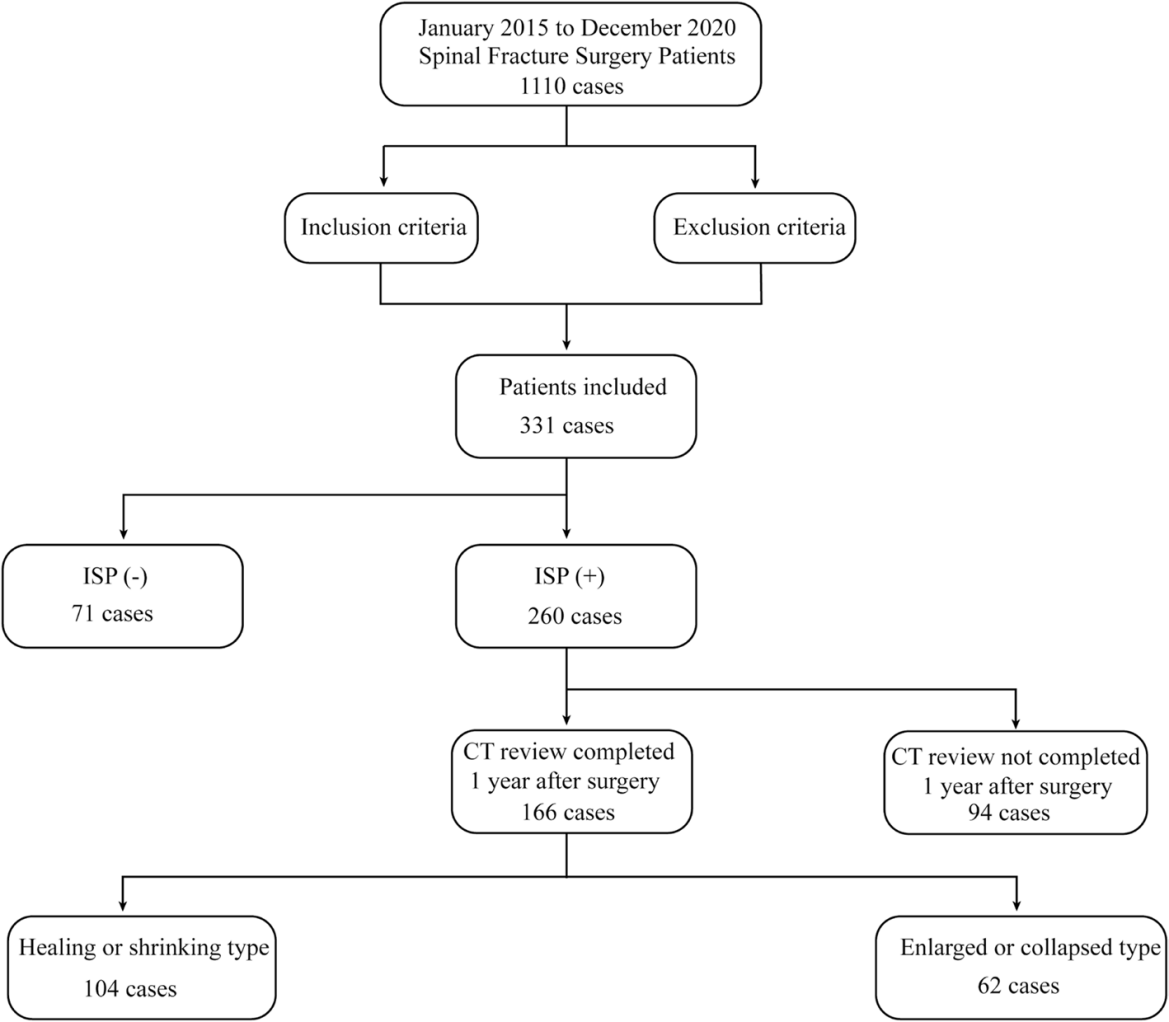
Data sources and selection. From January 2015 to December 2020, a total of 1,110 patients completed surgery. A total of 331 patients were included in the study through screening by inclusion and exclusion criteria. A total of 166 patients with ISP completed a CT review 1 year after surgery. Then the review results of these 166 patients were studied, of which 104 patients had a reduced or healed cavity and 94 had an enlarged cavity or adjacent tissue damage

Patients with fractures of the thoracolumbar spine (T11 to L2) who were admitted to the Department of Spine Surgery of the University Hospital between January 2015 and December 2020 were included in this retrospective study (Fig. 1). The patient inclusion criteria were as follows: (1) single-segment Denis compression and burst fractures of T11-L2 within 2 weeks after injury [12]; (2) combined or uncombined posterior ligamentous complex injury and intradiscal occupancy with minor or no neurological injury; and (3) perfect imaging data, including preoperative and postoperative vertebral body CT scans, postoperative follow-up data after more than 1 year, and CT or X-ray review results. The exclusion criteria were as follows: (1) T11-L2 fracture with substantial subluxation or severe spinal nerve injury requiring anterior decompression and implant fusion; and (2) cranial or thoracoabdominal injury or other systemic disease precluding surgery or necessitating postponement of surgery.

## Observed indicators

In all, 331 patients with thoracolumbar fractures were included in this retrospective analysis. Three independent and unbiased physicians were involved in the collection and measurement of patient data. We collected basic information such as sex, age, duration of illness (time from injury to surgery), duration of hospitalization (time from admission to discharge), bone mineral density (BMD), mode of injury, mode of surgery, presence of concomitant neurological symptoms, and presence of concomitant chronic diseases. We collected preoperative and postoperative CT and X-ray images of the thoracolumbar segment. The observation and measurement parameters included the degree of preoperative vertebral compression, preoperative sagittal Cobb angle (kyphotic angle, KA), presence of combined spinal canal occupancy, presence of screw-induced vertebral injury, presence of pedicle screw reinforcement with bone cement, the degree of postoperative vertebral bracing, the degree of postoperative vertebral body repositioning, the screw positions, the depth of screw insertion and the number of screws.

## Measurement methods

**Fig. 2:**
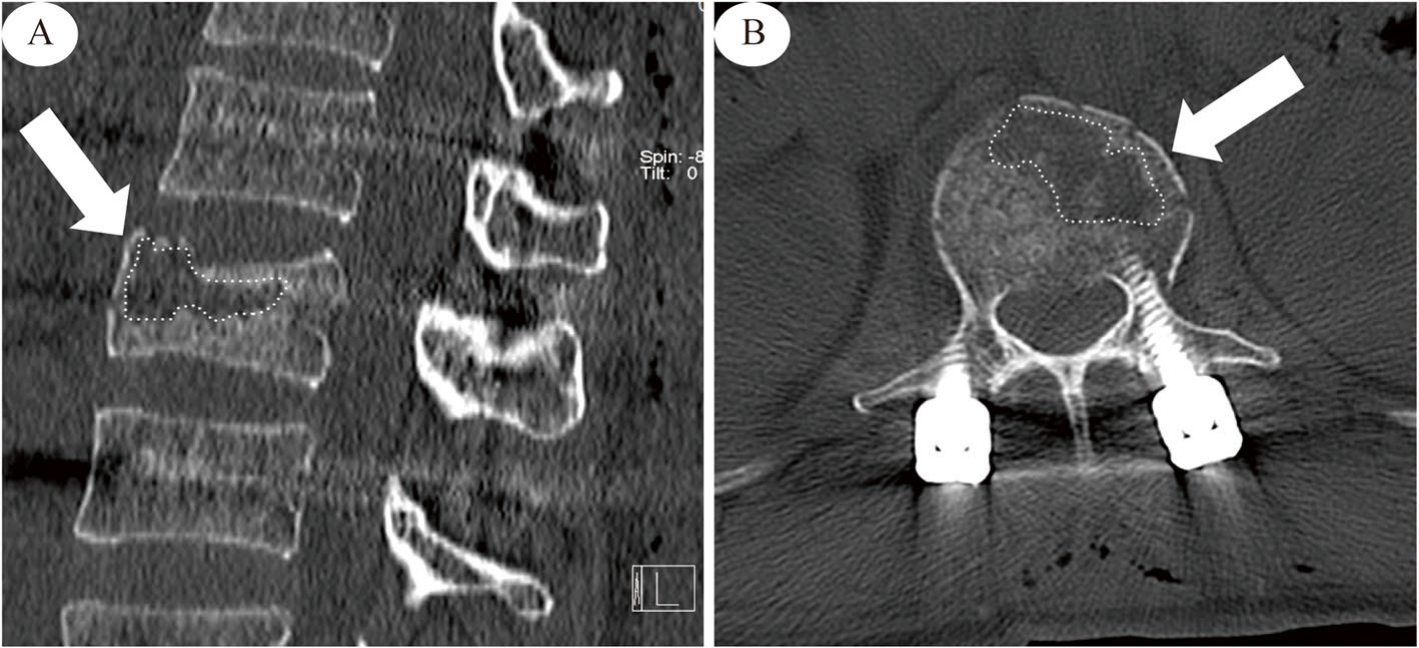
ISP Diagram. **A** CT sagittal view, the area indicated by the white arrow in the figure. **B** CT horizontal position, the area indicated by the white arrow in the figure

**Fig. 3:**
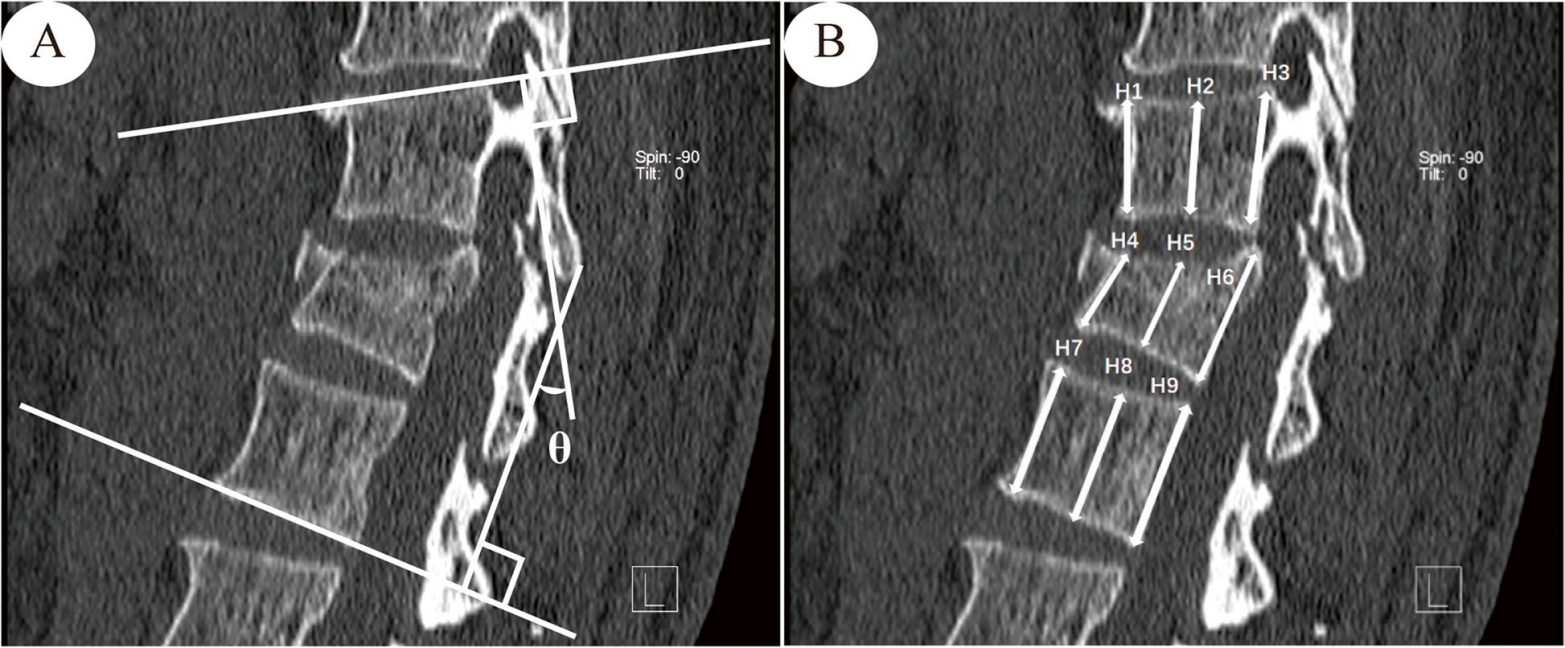
**A** Diagram of Cobb angle measurement in the sagittal plane of the vertebral body. **B** Diagram for measuring the degree of vertebral compression. UVH (Upper vertebral height) = ( H1 + H2 + H3) / 3. LVH (Lower vertebral height) = ( H7 + H8 + H9) / 3. IVH (Injured vertebra height) = ( H4 + H5 + H6) / 3. VBCR (Vertebral compression ratio) = IVH / [ (UVH + LVH ) / 2 ]

(1) ISP: ISP was defined as a low-density vacuum eggshell-shaped area in the vertebral body on postoperative sagittal or horizontal CT images (Fig. 2). (2) Vertebral compression ratio (VBCR): The VBCR was defined as (injured vertebral height [IVH])/[(upper vertebral height [UVH]) + (lower vertebral height [LVH])/2] × 100%, with the vertebral body height measured as the average of the anterior, middle, and posterior height (Fig. 3). (3) Sagittal Cobb angle: According to Phillips et al [13], the Cobb angle was defined as the angle at which the extension of the superior endplate of the injured vertebral body intersected the inferior endplate of the inferior vertebral body (Fig. 3). (4) Degree of vertebral body spreading: If the height of the anterior edge of the intervertebral space was greater than or equal to the height of the next intervertebral space, the lesion was considered overspread, and if the height of the anterior edge of the vertebral space was less than the height of the next vertebral space, the degree of spreading was considered moderate. (5) Degree of vertebral body repositioning: The height of the injured vertebra was taken as the average of the sum of the heights of the adjacent upper and lower vertebrae and considered excellent when > 90%; good, 80%-90%; and poor, < 80%. (6) Screw position: The screw position was evaluated by whether the direction of nail placement was parallel to the upper endplate. (7) Screw insertion depth: The screw insertion depth into the anteroposterior diameter of the vertebral body was measured on sagittal CT images. (8) BMD testing: A HOLOGIC GE dual-energy X-ray bone densitometer was used to examine BMD. The WHO [14] developed diagnostic criteria using the standard deviation (SD) for BMD values related to the peak bone mass in healthy young women, with osteoporosis defined as a BMD T score of -2.5 or lower and low bone mass (osteoporosis) defined as a BMD T score between − 1 and − 2.5. In conjunction with related studies [15], severe osteoporosis was defined as a mineral density T score of less than − 2.5 and a history of fragility fracture.

### Statistical methods

All statistical analyses were performed using SPSS statistical software (version 27.0; IBM Corporation, Armonk, NY). *P* values less than 0.05 were considered to indicate statistical significance. The assignment of the relevant variables is detailed in the Supplementary Materials (Supplementary Materials Table S1). Directed acyclic graphs (DAGs) illustrating the selection of potential risk factors are shown in Supplementary Material Figure S1. Univariate logistic regression analysis was used to evaluate the associations between each variable and the dependent variable, and the significant factors were subsequently included in the multivariate logistic regression analysis. Finally, the most significant influencing factors were screened.

## Results

### General information

A total of 331 patients (141 males and 190 females) with thoracolumbar spinal fractures treated with internal fixation surgery were included in this study. The mean age was 62 years (range, 21–85 years), the mean time from injury to surgery was 5 days (range, 1–14 days), and the mean number of hospital days was 16 days (range, 6–96 days). A total of 238 (71.9%) Denis fractures were classified as compression fractures, including 15 type A, 90 type B, 16 type C, and 117 type D fractures. There were 93 cases (28.1%) of burst fractures, including 30 type A, 29 type B, 4 type C, 30 type D, and 0 type E fractures. The average preoperative vertebral compression degree was 27% (range, 3–74%). The average postoperative degree of vertebral body repositioning was 91% (range, 54–98%). The average preoperative sagittal Cobb angle was 12° (range, 0.6°-32.7°). The mean bone density was − 2.8 SD (range, 0- -6.92 SD).

### Occurrence of postoperative ISP

**Fig. 4:**
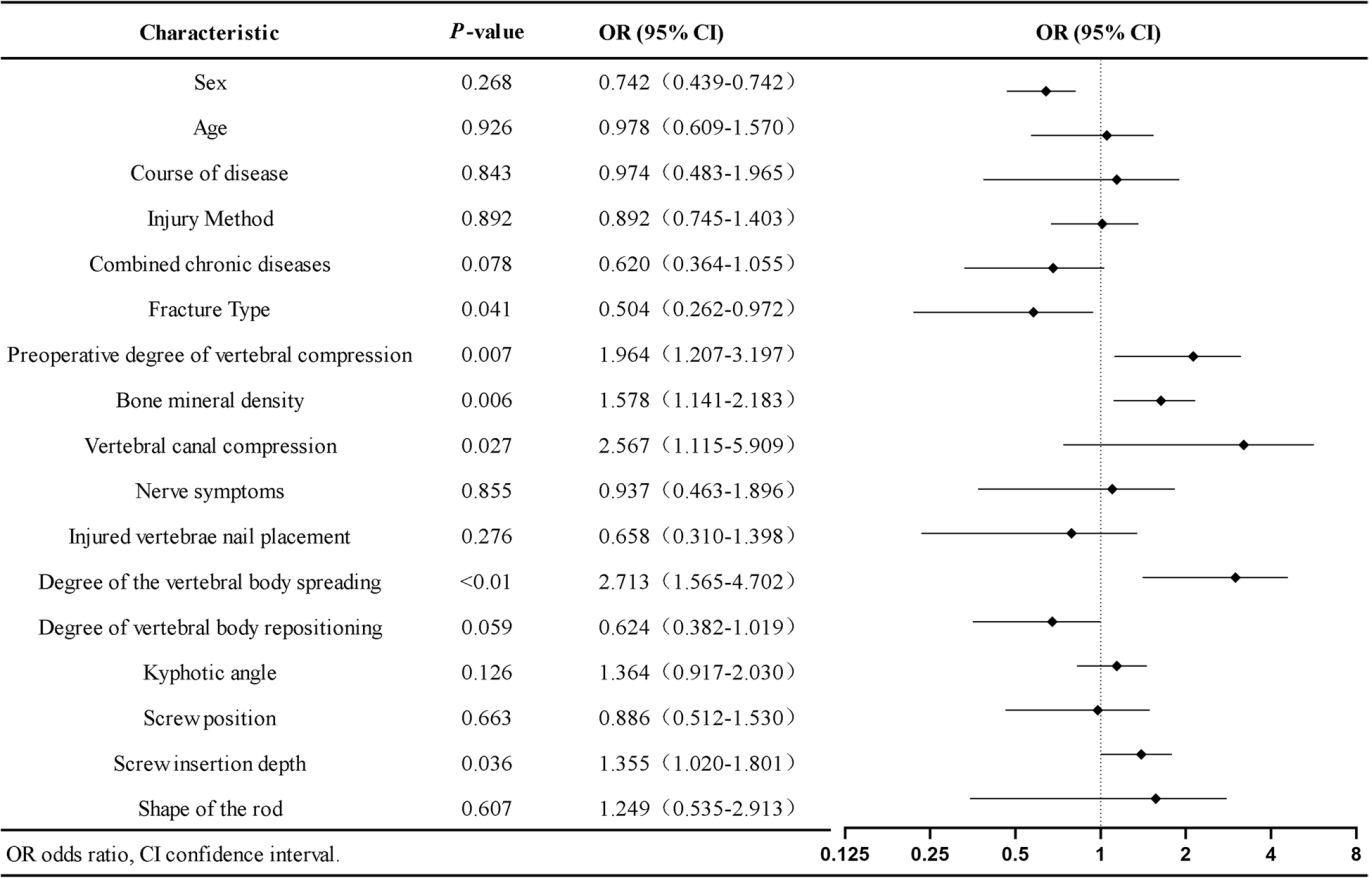
Post-surgical ISP univariate logistic regression

**Fig. 5:**
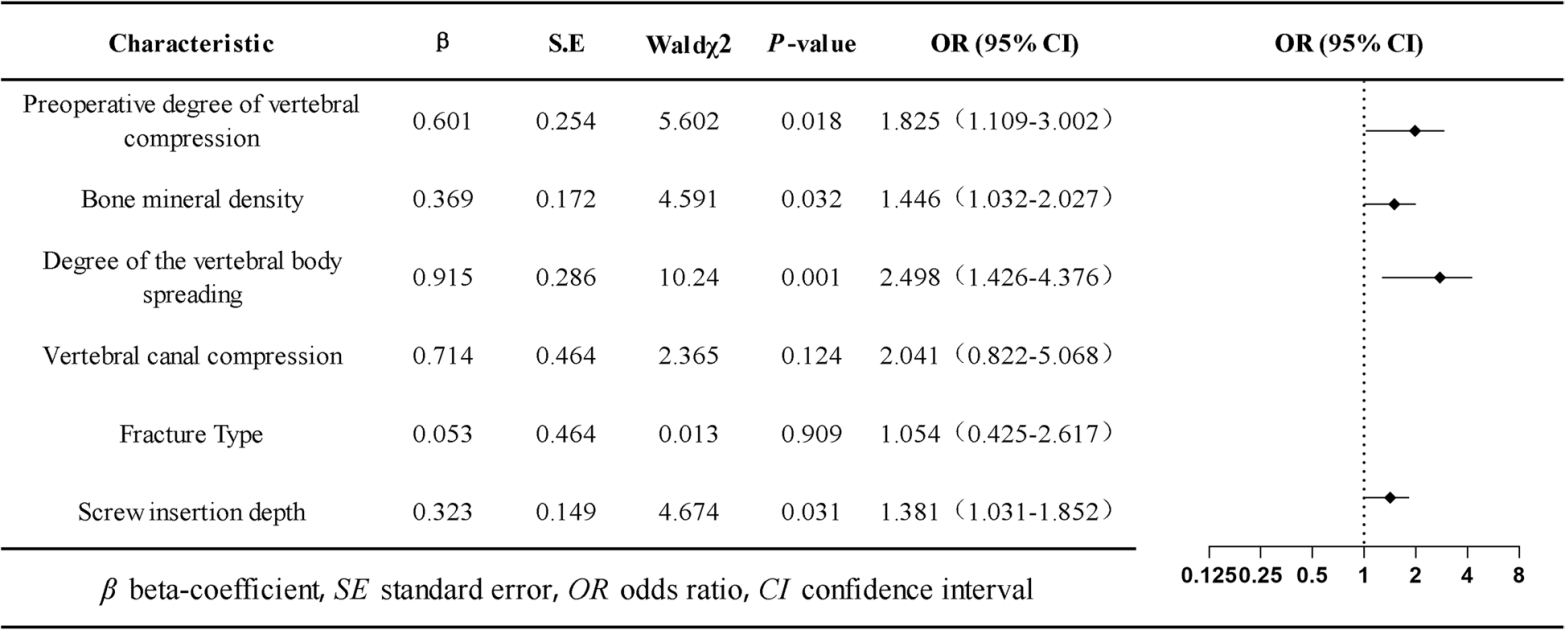
Post-surgical ISP multivariate logistic regression

The distribution of ISP in the 331 patients is shown in the Supplementary Materials (Supplementary Materials Table S2). In all, 260 of the 331 patients (78.5%) presented with postoperative ISP. Univariate logistic regression analysis revealed that bone density (*P* = 0.006), degree of preoperative vertebral compression (*P* = 0.007), degree of vertebral spreading (*P* ≤ 0.01), spinal canal occupancy (*P* = 0.027), fracture type (*P* = 0.041), and screw insertion depth (*P* = 0.036) were significantly associated with the occurrence of postoperative ISP (Fig. 4). Further multifactorial logistic regression analysis revealed that bone density (odds ratio [OR], 1.446; 95% confidence interval [CI], 1.032–2.027; *P* = 0.032), degree of preoperative vertebral compression (odds ratio [OR], 1.054; 95% confidence interval [CI], 1.109–3.002; *P* = 0.018), screw insertion depth (odds ratio [OR], 1.381; 95% confidence interval [CI], 1.031–1.852; *P* = 0.031), and intraoperative degree of vertebral body spreading (odds ratio [OR], 2.498; 95% confidence interval [CI], 1.426–4.376; *P* = 0.001) were significantly associated with postoperative ISP (Fig. 5).

### Evolutionary outcomes of ISP 1 year after surgery

**Fig. 6:**
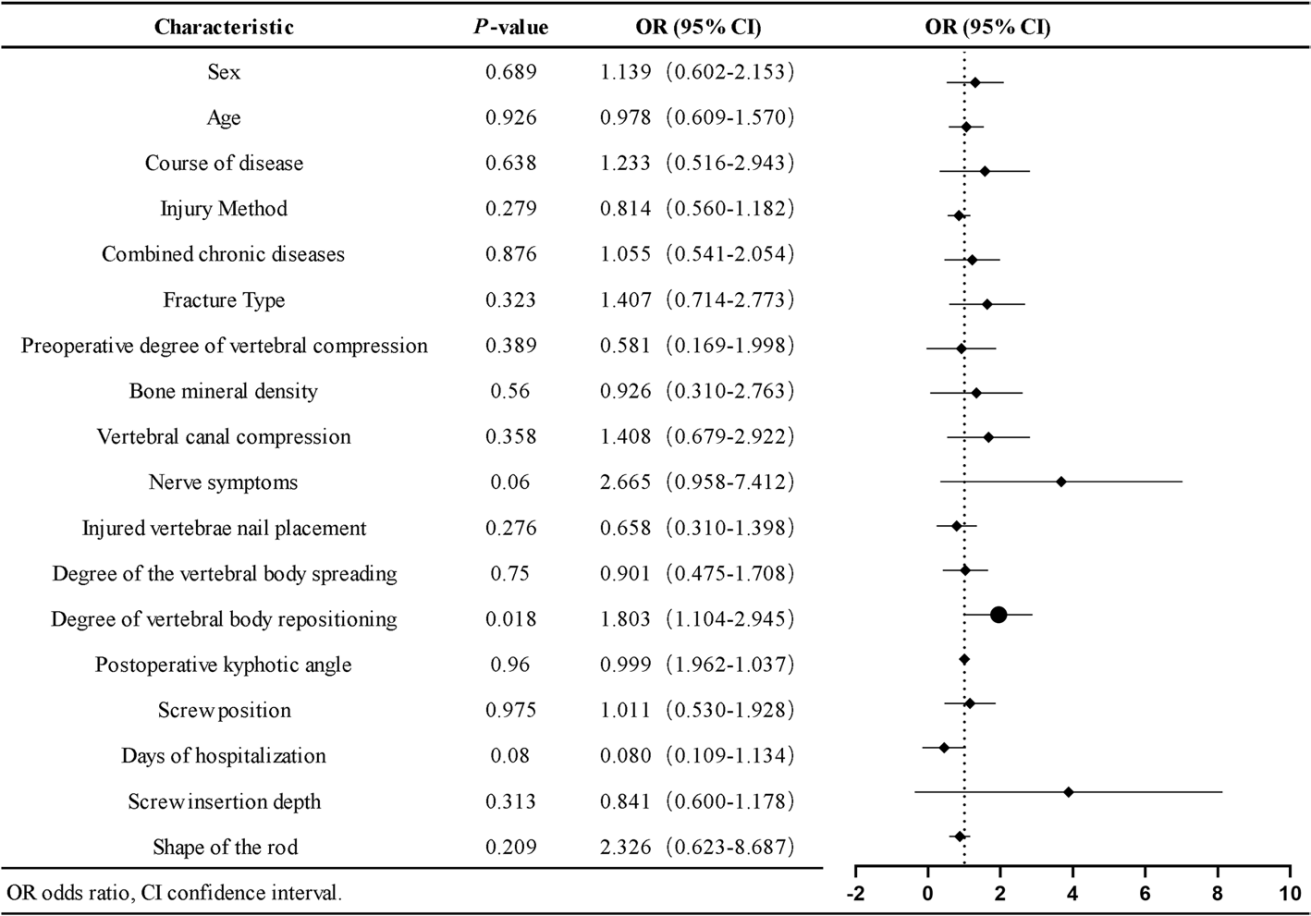
Univariate logistic regression of ISP evolutionary outcomes at 1 year postoperatively

A total of 166 patients were included in the study of the evolutionary outcomes of ISP 1 year after surgery. A total of 104 patients (62.6%) exhibited shrunken or healed cavities, and 62 patients (37.4%) exhibited enlarged cavities or collapsed endplates. Univariate and multifactorial logistic regression analyses showed that the degree of postoperative vertebral body repositioning (odds ratio [OR], 2.143; 95% confidence interval [CI], 1.243–3.696; *P* = 0.006) had a statistically significant effect on the 1-year postoperative ISP evolutionary outcome (Fig. 6).

## Discussion

Thoracolumbar fractures are the most common type of spinal fracture and are often classified as compression or burst fractures. Unlike limb fractures, vertebral fractures of the spine are usually accompanied by destruction of the trabeculae within the vertebral body. In routine surgical treatments, normally, only the height of the vertebral cortical bone can be restored, not the trabecular bone, leading to a residual cleft in the vertebral body. Several studies have shown that this phenomenon increases the risk of vertebral instability, which can lead to postoperative vertebral collapse, pedicle screw loosening, rod fracture, and loss of vertebral height, in turn leading to kyphosis and even serious complications such as posterior fusion failure, delayed healing or nonhealing of the vertebral body, and chronic low back pain [16–19].

Decreased BMD is one of the major causes of ISP. In this retrospective study, patients with osteopenia and severe osteoporosis were much more likely to develop ISP than patients with normal or reduced bone mass. The main reason for this difference may be the decreased bone density and loose bone within the vertebral body, leading to disorganization of the trabecular bone arrangement within the vertebral body after injury. After the vertebral body is fixed with a screw-rod system, although the shape is basically restored, the bone trabeculae within the vertebral body are still in a disordered state and cannot effectively brace the vertebral body. Further enlargement of the cavity encased by eggshell-shaped bone, coupled with localized ischemic necrosis, prevents repair of the bone defect in the vertebral body and closure of the cavity, leading to delayed healing or nonunion of the fracture. If the patient is unable to perform strict spinal restraint after surgery, due to the separated fracture fragments of the injured vertebral body and the mutual friction of the small vertebral joints, the pressure and temperature changes in the cavity of the injured vertebra may allow the cavity to be filled with gas or liquid, forming a vacuum in the vertebral body and the intervertebral disc [20–22]. In addition, changes in BMD caused by other factors are equally noteworthy, e.g., age, sex, and hormonal changes.

Among the common surgical options for the treatment of thoracolumbar spinal fractures, screw-rod internal fixation systems usually utilize the tension of the posterior longitudinal ligament and the fibrous annulus adjacent to the vertebral body for bracing; although the vertebral body can be immobilized and corrected to restore its normal shape of the vertebral body, full restoration of its endosteal structure is unlikely [23]. Theoretically, it is possible to restore the normal height of the injured vertebra, but if the vertebral body is forcibly propped up and the shape of the vertebral body is excessively restored, this can lead to further enlargement of the injured vertebral cavity and increase the difficulty of vertebral healing. In addition, if the KA measured via X-ray is too large, the biomechanical lines of force at the injured vertebral body will likewise be altered, affecting the alignment of the vertebral body on the sagittal plane and subsequent fracture repair. Wei-En Hsu et al. [24] found that when the vertebral body height loss or KA measured on CT was greater, the probability of finding an intravertebral cleft was higher. However, the effect of the KA on ISP was not statistically significant in this study. Further studies are still needed to prove the clinical significance of the effect of the KA on ISP.

We categorized spinal fractures into burst and compression fractures according to the Denis classification for inclusion in this study. In contrast to compression fractures, burst vertebral fractures are more destructive, involve more discrete fracture fragments and are associated with a greater chance of ISP after surgery. In clinical management, it is essential to consider whether to apply bone cement or autologous bone in the treatment of patients with burst fractures. In addition, the data from this study suggest that screw placement in the injured spine decreased the incidence of ISP, although the difference was not statistically significant. Previous studies have shown that the placement of pedicle screws in the injured vertebral segment increases the stiffness of the internal fixation system, thereby reducing the incidence of internal fixation device failure, screw pullout, and progressive deformity and increasing the overall stability of the spine [3, 25]. This could be related to the fact that the screws reduce the micromotion of fractures and fragments; however, additional confirmation from follow-up studies is needed. In addition, the depth of screw insertion is an important influencing factor of ISP. Shin Oe et al. [26] reported that increasing the screw length to 81.8% or more of the vertebral body reduced the load on the upper instrumented vertebra, thus enhancing the stability of the internal fixation system. This observation also supports our findings.

**Fig. 7:**
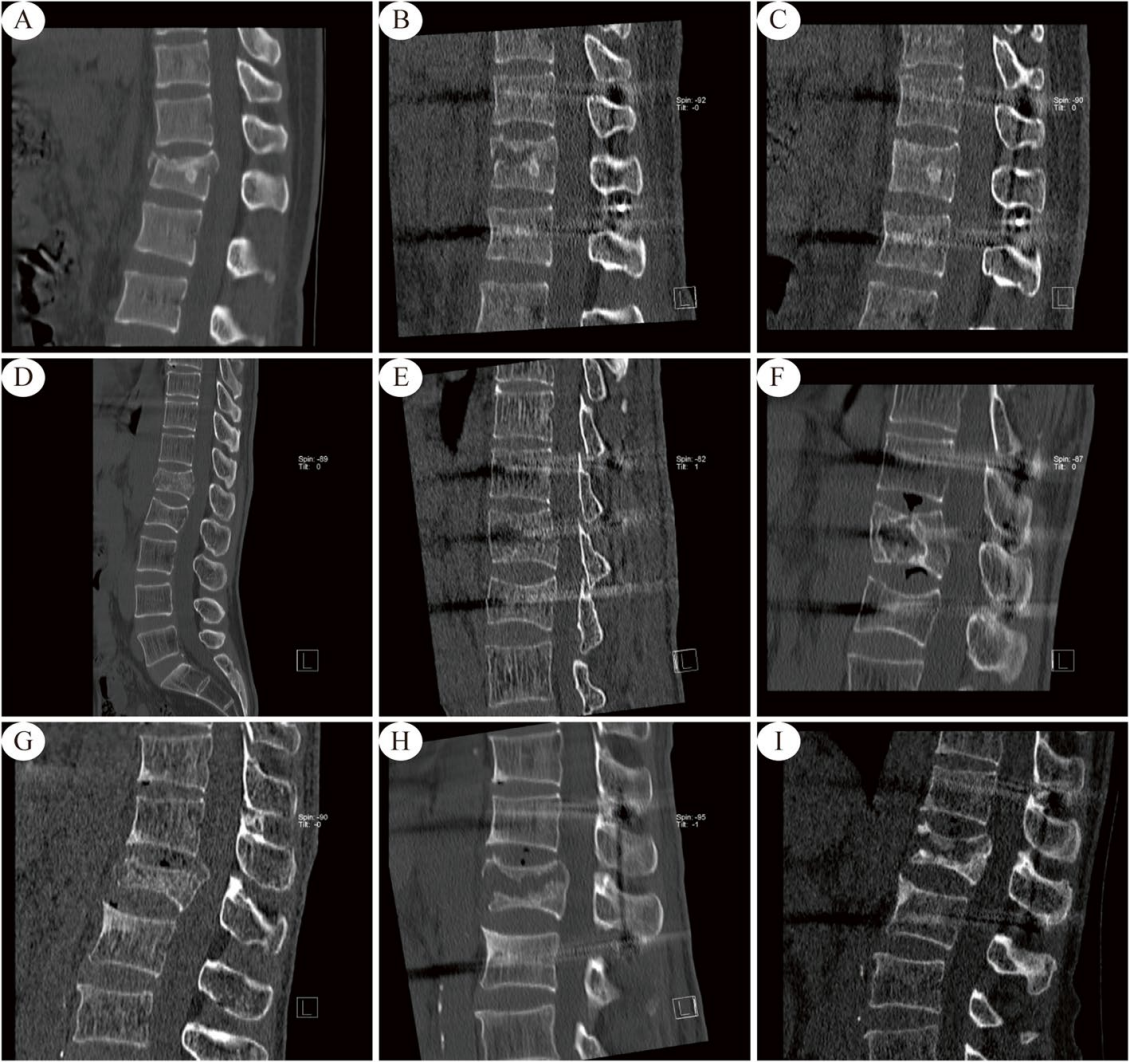
The preoperative, 3 days postoperative and 1-year postoperative CT sagittal views are shown from left to right. **A**, **B**, **C** Shrinkage or healing type. Female ,36 years. L1 Compression fracture. Traffic Accidents. **D**, **E**, **F** Enlarged or end plate collapse type. Male,49 years. L1 Burst fracture. High fall injuries. **G**, **H**, **I** Shrinkage with endplate collapse type. Male ,62 years. L1 Burst fracture. High fall injurie

At 1 year after surgery, we review follow-up CT images of patients with associated ISP. The results of the present study showed that patients with a greater degree of postoperative vertebral repositioning achieved better healing of the injured vertebral cleft at 1 year postoperatively. The reason for this finding may be that the closer the height of the injured vertebra is to the normal physiologic height, the greater the support provided for the vertebra and adjacent structures. According to our CT imaging observations, the evolutionary outcomes at 1 year after posterior internal fixation for thoracolumbar fractures could be classified into the following two categories: reduced volume or complete healing of the vertebral body cavity; or expansion of the vertebral body cavity with invasion of the adjacent endplate, resulting in endplate collapse and disc invagination (Fig. 7) (Supplementary Materials Figure S2). In addition, in some cases, the vertebral cavity shrank 1 year after surgery, but the adjacent endplate collapsed (Fig. 7); we classified such cases as shrinking or healing. Of course, this outcome does not exclude the possibility of subsequent cavity enlargement, which requires longer follow-up observation.

In the surgical management of spinal fractures, ISP is a problem that clinicians cannot completely avoid. ISP is involved in the entire process from the beginning of the injury to complete healing of the fracture and poses a challenge to the patient's prognosis. ISP can lead to instability and failure of internal fixation devices, breakage of adjacent endplates and discs, and chronic back pain. Internal fixation in combination with other treatment modalities is necessary to minimize the occurrence of ISP during initial treatment [27, 28]. Examples of such modalities include cement reinforcement of the injured vertebra and filling with other bone materials [29, 30]. The surgeon not only needs to know the patient's BMD and the extent of the spinal injury but also moderately spread compressed vertebrae to restore as normal a physiologic height as possible.

### Limitations

As this was a retrospective study, the results must be interpreted with caution. Despite having three independent and unbiased observers, the problem of high ISP rates could not be avoided and may lead to bias in the results. In addition, the study lacked a conservative treatment control, and the occurrence of ISP after nonsurgical treatment for these fractures requires further study. We studied the changes in ISP at 1 year after surgery, which has some limitations in terms of the inability to observe the ultimate outcome. However, due to the retrospective design of this study, it was not possible to fully incorporate factors such as patients' other diseases and symptoms to determine the long-term effects of ISP on the patient.

## Conclusion

Regarding the effect of ISP on postoperative thoracolumbar fracture patients, clinicians need to pay special attention to the BMD, vertebral body height, screw insertion depth, and degree of vertebral repositioning. ISP persists in some patients 1 year after surgery and may lead to internal fixation instability, vertebral body nonhealing, and disc collapse.

### Supplementary Information

**Additional file 1.**

## Data Availability

The datasets used and/or analysed during the current study available from the corresponding author on reasonable request.
