# Supplementary material for: Analysis of factors influencing the intravertebral shell phenomenon after posterior reduction internal fixation of thoracolumbar fracture: a retrospective study
Source: BMC Musculoskelet Disord. 2024 Jan 10;25:49. doi: 10.1186/s12891-024-07168-9 (PMC10777656; doi:10.1186/s12891-024-07168-9)
Supplement: Supplementary file 1 — Additional file 1. [file 12891_2024_7168_MOESM1_ESM.docx]

Supplementary Content

Figure S1.Directed acyclic graphs (DAGs) illustrating the selection of potential risk factors.......................................................................2

Figure S2. Endplate collapse and disc herniation into the vertebral body

.....................................................3

Table S1. Assignment of relevant influencing factors.............................4-5

Table S2. Distribution of ISP in patients....................................................6


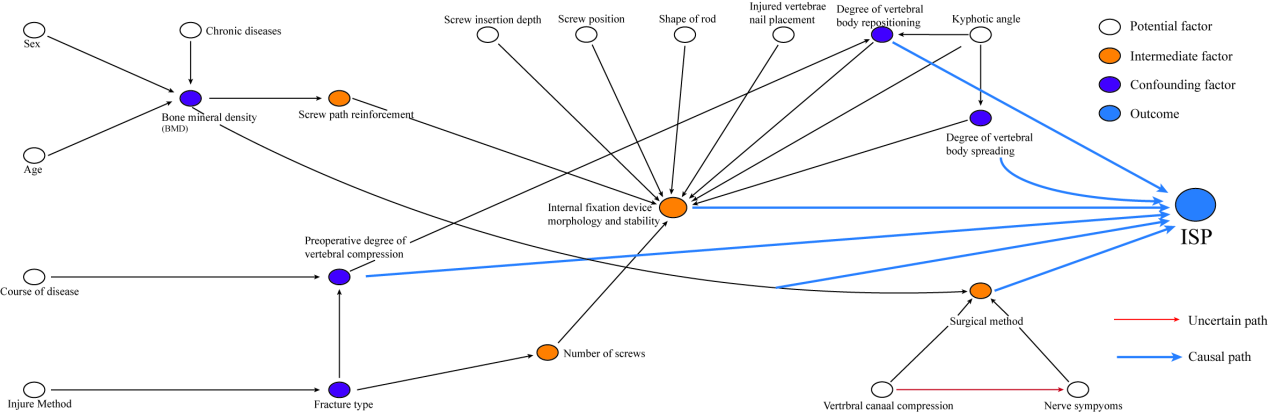


**Figure S1 Directed acyclic graphs (DAGs) illustrating the selection of potential risk factors**

The potential risk factors assessed include all factors other than those excluded from the outcome in the figure above. Among these, screw channel reinforcement, internal fixation device morphology and stability, and surgical approach were intermediate factors (such factors may be influenced by a combination of factors that can have a causal effect on the outcome). In addition, BMD, preoperative vertebral compression, fracture type, degree of vertebral repositioning, and degree of vertebral support were confounding factors (such factors are jointly influenced by a variety of other factors, thus forming a causal relationship on the outcome, but they can also directly influence the outcome). We excluded intermediate factors and included confounders for logistic regression analysis. For vertebral canal occupation and neurologic symptoms, there was no definite causal relationship between them, so both were included in the factor analysis.


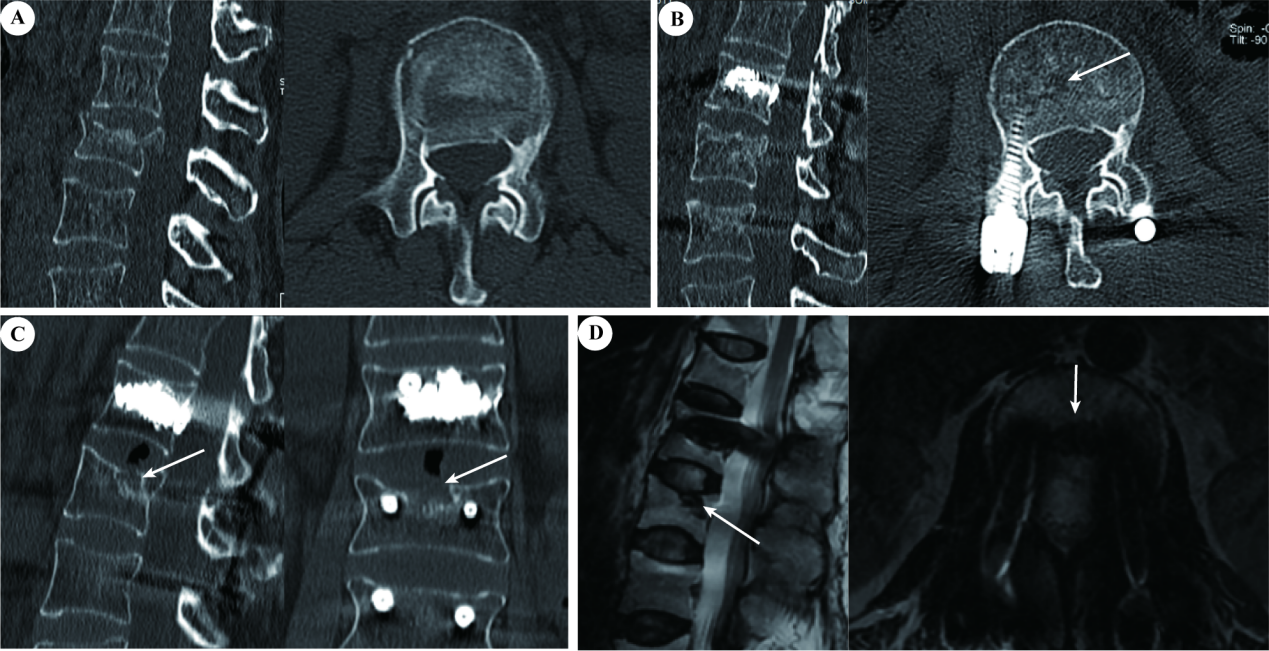


**Figure S2 Endplate collapse and disc herniation into the vertebral body.**

Female, 51 years. L2 Burst fracture. High fall injuries. **(A)** Preoperative CT horizontal and sagittal views. **(B)** Three days postoperative CT sagittal and horizontal positions. **(D)** 15-month postoperative CT sagittal and coronal position, white arrows show endplate collapse and disc herniation into the vertebral body. **(D)** 15-month postoperative MRI sagittal and horizontal views, the white arrow shows the disc herniated into the tissue, which is seen to be the same density as the tissue within the disc and vertebral body.

Table S1 Assignment of relevant influencing factors

| **Characteristic** | **Assignment** | |
| --- | --- | --- |
| Intravertebral shell phenomenon | No | 0 |
|  | Yes | 1 |
| Sex | Female | 0 |
|  | Male | 1 |
| Age | 18-40 | 0 |
|  | 41-60 | 1 |
|  | >60 | 2 |
| Course of disease  (Time from injury to surgery) | No | 0 |
|  | Yes | 1 |
| Injury Method | Impact injury | 0 |
|  | Traffic Accidents | 1 |
|  | Falling from a height | 2 |
| Combined chronic diseases  (E.g. high blood pressure, diabetes, heart disease, etc.) | No | 0 |
|  | Yes | 1 |
| Surgical method | PPSF | 0 |
|  | ORIF | 1 |
| Fracture Type | Compression fracture | 0 |
|  | Burst fracture | 1 |
| Preoperative degree of vertebral compression | ＜25% | 0 |
|  | 25%-50% | 1 |
|  | 51%-75 | 2 |
|  | ＞75%=3 | 3 |
| Bone density | Bone mass normal | 0 |
|  | Bone mass loss | 1 |
|  | Osteoporosis | 2 |
|  | Serious osteoporosis | 3 |
| Vertebral canal compression | No | 0 |
|  | Yes | 1 |
| Nerve symptoms | No | 0 |
|  | Yes | 1 |
| Injured vertebrae nail placement | No | 0 |
|  | Yes | 1 |
| Degree of vertebral body spreading | Moderate distraction | 0 |
|  | Excessive distraction | 1 |
| Degree of vertebral body repositioning | Excellent | 0 |
|  | Good | 1 |
|  | Poor | 2 |
| Preoperative kyphotic angle | <=10° | 0 |
|  | 10°-20° | 1 |
|  | >20° | 2 |
| Postoperative kyphotic angle | <0° | 0 |
|  | 0°-10° | 1 |
|  | >10° | 2 |
| Screw position | Not Parallel to the upper end plate | 0 |
|  | parallel to the upper end plate | 1 |
| Screw path reinforcement ^†^ | No | 0 |
|  | Yes | 1 |
| Number of screws | <=4 | 0 |
|  | 5-7 | 1 |
|  | >=8 | 2 |
| Days of hospitalization  (Time from start of hospitalization to discharge) | <=14days | 0 |
|  | 15-21days | 1 |
|  | >=22days | 2 |
| Evolutionary outcomes of ISP 1 year after surgery | Shrink or heal | 0 |
|  | Enlargement or collapse | 1 |
| Removal of internal fixation after 1 year | Yes | 0 |
|  | No | 1 |
| Screw insertion depth^*^ | >80% | 0 |
|  | 70%-80% | 1 |
|  | 60%-70% | 2 |
|  | <60% | 4 |
| Shape of the rod  (Does the titanium rod fit the curvature of the spine) | Does not fit the curvature of the spine | 0 |
|  | Fits the curvature of the spine | 1 |
| (PPSF) Minimally invasive percutaneous pedicle screw fixation.  (ORIF) Open reduction and internal fixation.  ^†^ Use bone cement reinforcement for screwed vertebral channels.  * Length of screws entering the vertebral body as a percentage of the anterior-posterior length of the vertebral body. | | |

**Table S2 Distribution of ISP in patients**

| **Characteristic** | **Level** | **ISP (-)** | **ISP (+)** |
| --- | --- | --- | --- |
| Cases |  | 71 | 260 |
| Age | 18-40 | 2 | 9 |
|  | 41-60 | 25 | 90 |
|  | >60 | 44 | 161 |
| Fracture Type | Compression fracture | A 2 | A 13 |
|  |  | B 26 | B 64 |
|  |  | C 5 | C 11 |
|  |  | D 25 | D 92 |
|  | Burst fracture | A 3 | A 27 |
|  |  | B 6 | B 23 |
|  |  | C 0 | C 4 |
|  |  | D 4 | D 26 |
|  |  | E 0 | E 0 |
| Fracture segment | T11 | 2 | 4 |
|  | T12 | 40 | 50 |
|  | L1 | 39 | 144 |
|  | L2 | 20 | 62 |
| Bone density | Bone mass normal | 12 | 22 |
|  | Bone mass loss | 26 | 53 |
|  | Osteoporosis | 24 | 161 |
|  | Serious osteoporosis | 9 | 24 |
| Preoperative degree of vertebral compression | ＜25% | 46 | 125 |
|  | 25%-50% | 24 | 117 |
|  | 51%-75% | 1 | 18 |
|  | ＞75% | 0 | 0 |
| kyphotic angle | <=10° | 37 | 109 |
|  | 10°-20° | 27 | 115 |
|  | >20° | 7 | 36 |
| Degree of vertebral body spreading | Moderate distraction | 47 | 105 |
|  | Excessive distraction | 24 | 151 |
| Injured vertebrae nail placement | No | 60 | 232 |
|  | Yes | 11 | 28 |
| Degree of vertebral body repositioning | Excellent | 55 | 169 |
|  | Good | 13 | 70 |
|  | Poor | 3 | 21 |
| ISP(+) Vertebrae with shell phenomenon.ISP(-) Vertebrae without shell phenomenon. | | | |
